# Evaluation of fluorescence in situ hybridisation (FISH) for the detection of fungi directly from blood cultures and cerebrospinal fluid from patients with suspected invasive mycoses

**DOI:** 10.1186/s12941-015-0065-5

**Published:** 2015-01-31

**Authors:** Roberto Moreira Da Silva, João Ricardo Da Silva Neto, Carla Silvana Santos, Hagen Frickmann, Sven Poppert, Kátia Santana Cruz, Daniela Koshikene, João Vicente Braga De Souza

**Affiliations:** Universidade do Estado do Amazonas, Manaus, Brazil; Fundação de Medicina Tropical Dr. Heitor Vieira Dourado, Manaus, Brazil; Department of Tropical Medicine at the Bernhard Nocht Institute, German Armed Forces Hospital of Hamburg, Hamburg, Germany; Institute of Medical Microbiology, Justus-Liebig-University Giessen, Giessen, Germany; Instituto de Criminalística, Manaus, Brazil; Instituto Nacional de Pesquisas da Amazônia, Manaus, Brazil; Biotecnólogo/Tecnologista Pleno III, Instituto Nacional de Pesquisas da Amazônia, Coordenação de Sociedade, Ambiente e Saúde, Laboratório de Micologia, Av. André Araújo, 2936, Aleixo, Manaus, AM CEP 69060-001 Brazil

**Keywords:** FISH, Invasive mycoses, CSF, Blood culture, rRNA, Hybridisation

## Abstract

The aim of this study was to evaluate the diagnostic performance of in-house FISH (fluorescence in situ hybridisation) procedures for the direct identification of invasive fungal infections in blood cultures and cerebrospinal fluid (CSF) samples and to compare these FISH results with those obtained using traditional microbiological techniques and PCR targeting of the ITS1 region of the rRNA gene. In total, 112 CSF samples and 30 positive blood cultures were investigated by microscopic examination, culture, PCR-RFLP and FISH. The sensitivity of FISH for fungal infections in CSF proved to be slightly better than that of conventional microscopy (India ink) under the experimental conditions, detecting 48 (instead of 46) infections in 112 samples. The discriminatory powers of traditional microbiology, PCR-RFLP and FISH for fungal bloodstream infections were equivalent, with the detection of 14 fungal infections in 30 samples. However, the mean times to diagnosis after the detection of microbial growth by automated blood culture systems were 5 hours, 20 hours and 6 days for FISH, PCR-RFLP and traditional microbiology, respectively. The results demonstrate that FISH is a valuable tool for the identification of invasive mycoses that can be implemented in the diagnostic routine of hospital laboratories.

## Introduction

The number of invasive fungal infections has increased over the last few decades. This phenomenon is the result of the growing number of pathological or iatrogenic immunocompromising conditions, premature births, neoplasms, abdominal surgeries, medical device insertion procedures and antibiotic therapies [1,2].

In northern Brazil, specifically in the state of Amazonas, histoplasmosis and cryptococcosis are some of the most frequent causes of death in AIDS patients [3], and candidemia is a problem in neonatal intensive care units [4]. In northern Brazil, the current diagnostic procedures at the hospital laboratories for the detection and identification of invasive fungal infections include culture (blood cultures, clinical specimens cultured on selective fungal media), biochemical methods, microscopic morphological determination and immunological assays. However, these traditional methods are time consuming, and their sensitivity for early detection is low [5]. To overcome these limitations, molecular approaches can be used for the detection and identification of pathogenic fungi [6].

Molecular techniques, particularly PCR-based approaches, have been developed to detect fungi in a short period of time; these approaches include nested PCR, multiplex PCR, real-time PCR and microarray techniques [7]. Although they have shown convincing results, the assays remain expensive, and definitive results are guaranteed only after several hours of hands-on time by highly experienced microbiologists [7-9].

Fluorescence in situ hybridisation (FISH) has already been successfully implemented in clinical microbiology for the identification of various pathogens, including fungi [10-15]. The hybridisation of fixed fungi with fluorescently labelled oligonucleotide probes that are complementary to unique target sites on the ribosomal RNA allows direct microscopic visualisation without prior amplification steps, even from blood culture smears. As an alternative to DNA-based FISH probes, peptide nucleic acid (PNA) probes with a neutral backbone may be used, although these probes are considerably more expensive [10,12].

The literature contains several studies that have evaluated FISH for the detection and identification of Candida spp. [12,16,17] and for the detection of pathogenic Cryptococcus ssp. [14]. This study is the first demonstration of the application of FISH for the routine identification of the primary causative agents of invasive fungal infections in a diagnostic laboratory in South America. The aim of this study was to evaluate FISH for the identification of fungi directly from positive blood cultures and cerebrospinal fluid samples of patients with suspected invasive mycosis.

## Materials and methods

### Biological samples

Samples were collected from patients who had been referred to the Mycology Laboratory of the Fundação de Medicina Tropical Dr. Heitor Vieira Dourado, Manaus, Brazil (FMT-HVD) between November 2013 and April 2014. In total, 142 biological samples were investigated, including 112 cerebrospinal fluid (CSF) samples from patients with a clinical diagnosis of cryptococcosis and 30 blood cultures that had presented positive results (CO_2_ production) in a blood culture system BACTEC 9120 (Becton-Dickinson, Sparks, MD, USA); however, these blood samples were negative for bacteria according to Gram stain analysis. Anonymised patient information (sex, age, place of residence, clinical specimen investigated, HIV serology results, HIV viral load and CD4^+^ cell count) was obtained from the computerised system “iDoctor Hospital”, which is used at FMT-HVD.

### Detection and identification of pathogenic fungi by conventional methods

The detection and identification of pathogenic fungi were performed using traditional biochemical and micro-morphological identification methods as previously described [18]. Initially, the CSF samples were centrifuged (5000 × g per 15 min), and the supernatant (90% of the initial volume) was carefully removed. Slides for direct microscopic examination were prepared with samples (20–50 μL) of CSF following centrifugation (5000 × g, 5 min) and of blood cultures (without any previous treatment). India ink was used for the visualisation of Cryptococcus spp. capsules, and lactophenol cotton blue and 10% potassium hydroxide (KOH) were used for the visualisation of general fungal cells. Cultures from 100–200 μL samples (CSF and blood culture) were grown using Sabouraud agar (BD Difco, Sparks, USA) and Mycosel agar (BD Difco, Sparks, USA). The fungal cultures were inoculated on Niger seed agar and CHROMagar™ Candida selective media (BD Difco, Sparks, USA). When the selective media were insufficient for the determination of the pathogens’ species, the isolates were subsequently subjected to micro-morphological and physiological tests using the fungal identification kit API20 (bioMérieux Vitek, Inc., Hazelwood, MO, USA).

### Detection and identification of pathogenic fungi by PCR-RFLP

PCR product generation and subsequent digestion for RFLP analysis was performed as described by Santos [19]. DNA was extracted from 200 μL biological samples using a QIAamp Blood and Tissue Kit (Qiagen, Hilden, Germany) according to the manufacturer’s instructions. The DNA was quantified by absorbance at 260 nm using a GeneQuant spectrophotometer (Eppendorf, Hamburg, Germany). Twenty nanograms of the extracted DNA served as template for PCR amplification. PCR reactions were performed in a total volume of 25 μL containing PCR buffer (final concentration: 1x; 10 mM Tris–HCl (pH 8.3) and 50 mM KCl), 1.5 mM MgCl_2_, 200 nM of the primers ITS5 (5′-GGAAGTAAAAGTCGTAACAAGG-3′) and NL4 (5′-GGTCCGTGTTTCAAGACGG-3′) (both primers were described previously by Irobi et al. [20]), 50 μM dNTPs, and 1 U ampli-Taq DNA polymerase. PCR was performed using a thermocycler (Kyratec SuperCycler, Seoul, South Korea) with the following conditions: initial denaturation for 5 min at 94°C, followed by 40 cycles for 30 s at 94°C (denaturation), 30 s at 50°C (annealing), and 90 s at 72°C (extension), with a final extension for 10 min at 72°C. The PCR products were visualised by electrophoresis on a 2% agarose gel and stained with SYBR® Green (SYBR Safe DNA Gel Stain, Invitrogen, Carlsbad, CA, USA). A DNA Ladder Mix (SM0331, MBI Fermentas, St. Leon-Rot, Germany) served as the size marker. The positive PCR products were identified using RFLP. The amplicons were digested with 10 U of the restriction enzyme DdeI (Thermo Fisher Scientific, Vilnius, Lithuania) for 3 h at 37°C and subjected to electrophoresis as described above. The sizes of the PCR products and restriction fragments generated from the isolates were compared with the corresponding previously described nucleotide sequences [20].

### Detection and identification of pathogenic fungi by FISH

The initial FISH reaction was performed using the pan-fungal probe, and then specific probes for each fungal species were used when fungal structures were found. These probes were chosen because of their high specificity and because of the absence of cross-reactions with other fungal species. The details of each probe used in the present study are presented in Table 1. In addition to the probes, the samples were counterstained with DAPI (4′,6-diamidino-2-phenylindole-dihydrochloride).

**Table 1:** **Information regarding the probes used in the present study**

**Target microorganisms**	**Probes**	**Sequences (5′-3′)**	**Formamide content in hybridisation buffer (% v/v)**	**NaCl content in wash buffer (M)**	**Authors**
C. albicans	Caal*	GCCAAGGCTTATACTCGCT	30	0.112	Kempf [10]
C. glabrata	Cagl*	CCG CCA AGC CAC AAG GAC T	30	0.112	Kempf [10]
C. parapsilosis	Capa*	CCTGGTTCGCCAAAAAGGC	20	0.225	Kempf [10]
Aspergillus spp.	Asp*	TGATACATTCCGAG	25	0.159	Wang [21]
C. neoformans and C. gattii	Cne205*	CCAGCCCTTATCCACCGA	20	0.225	Martins [14]
H. capsulatum	Hca1*	AGTCGAGGCTTTCAGCATGT	30	1.112	Silva Jr [22]
Fungi	Pan fungal**	CTCTGGCTTCACCCTATTC	30	0.112	Amann [23]

*Oligonucleotide probe synthesised and directly 5′-labelled with the hydrophilic sulphoindocyanine fluorescent dye Cy3 (Thermo Hybaid, Ulm, Germany).

**Oligonucleotide probe synthesised and directly 5′-labelled with fluorescein isothiocyanate (FITC) (Thermo Hybaid, Ulm, Germany).

The CSF samples were centrifuged at 10000 × *g* for 5 min at room temperature. The pellet containing cells was washed with 500 μL of phosphate-buffered saline (PBS; 130 mM sodium chloride and 10 mM sodium phosphate buffer (pH 7.2)) and fixed for 4 h with 4% w/v paraformaldehyde in PBS at 4°C. After fixation, the cells were washed twice with PBS, suspended in one volume of PBS and one volume of cold absolute ethanol and stored at −20°C until use.

For the investigation of pre-incubated blood cultures, 0.5 ml acetic acid (100%) was added to 5 ml blood culture medium to lyse the erythrocytes. The suspension was centrifuged at 10000 × *g* for 5 min. Then, the supernatant was discarded, and the pellet containing cells was treated following the same procedures described for treating the pellet obtained with CSF samples.

The FISH assay was performed as described by Amann [24]. The whole fixed cells were smeared onto precleaned microscopic slides and dried at 37°C for 20 min. Next, the slides were covered with hybridisation buffer (0.9 M NaCl, 0.01% w/v SDS, 20 mM Tris–HCl (pH 7.2), formamide and 1 μM probe) and incubated at 46°C for 2 h. After this period, the slides were washed with wash buffer (20 mM Tris–HCl (pH 8.0), 0.01% w/v SDS, 5 mM EDTA and NaCl) for 30 min at 46°C. The concentrations of formamide and NaCl in the hybridisation buffer and wash buffer, respectively, varied according to the probe (Table 1). Then, the slides were dried at 37°C for 20 min, mounted in Vectashield solution (Vector, Burlingame, CA, USA) and examined using a Zeiss Axioskop 40 microscope (Zeiss, Jena, Germany).

### Ethical considerations

Ethical clearance for this study was obtained from the Ethical Committee at the Fundação de Medicina Tropical Heito Viera Dourado in accordance with Brazilian laws relating to research with human subjects.

## Results

Conventional microscopy (India ink) and FISH were performed to confirm or exclude the presence of fungal agents in 112 CSF samples from patients with the clinical suspicion of cryptococcosis. Table 2 displays the calculated sensitivities and specificities of the culture, conventional microscopy and FISH analyses, using PCR for detecting yeast from the Cryptococcus neoformans complex, which includes C. neoformans and C. gattii, in cerebrospinal fluid as a reference. In total, 46 CSF samples tested positive by both conventional microscopy and FISH. In contrast, 2 samples tested positive by FISH and PCR but negative by conventional microscopy.

**Table 2:** **Sensitivity and specificity of culture, microscopy and FISH analyses as calculated using PCR as the gold standard for the detection of C. neoformans and C. gattii in CSF**

			**PCR**	
			**Positive**	**Negative**
**Tests compared to PCR**	**FISH**	Positive	48	0
		Negative	0	64
	Sensitivity (%)		100	
	Specificity (%)		100	
	**Microscopy (Direct examination)**	Positive	46	0
		Negative	2	64
	Sensitivity (%)		95.8	
	Specificity (%)		100	
	**Culture**	Positive	41	0
		Negative	5	66
	Sensitivity (%)		89.1	
	Specificity (%)		100	

**Figure 1:**
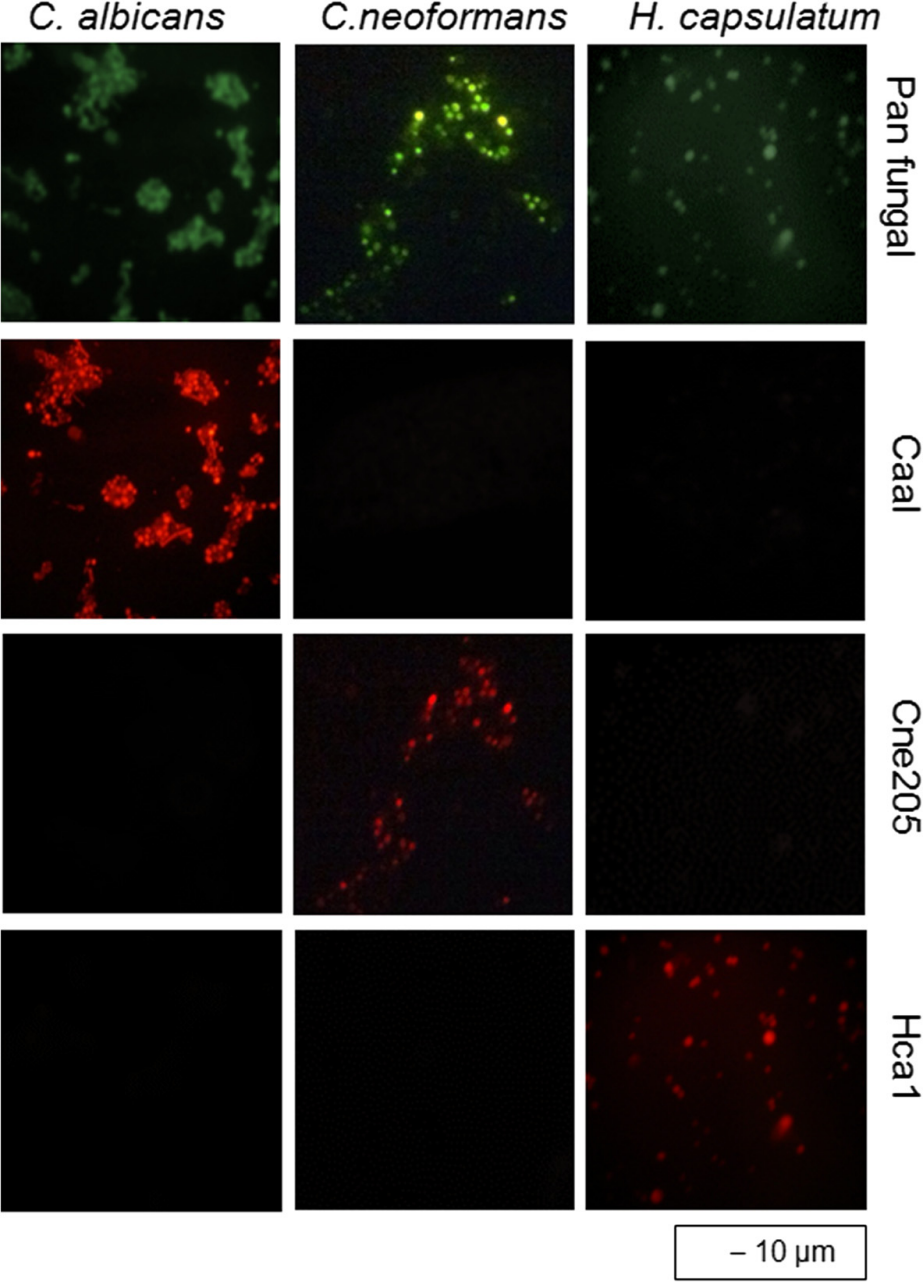
**Fluorescence microscopy of microorganisms after FISH with various oligonucleotide probes.** C. albicans, C. neformans and H. capsulatum (vertical columns) were stained with FISH with probes Pan Fungal (green signal; specific for all fungi) and Caal (specific for C. albicans), Cne 205 (specific for C. neoformans and C. gattii), and Hca1 (specific for H. capsulatum), each Cy3 labelled (red signal).

Thirty blood cultures were investigated, and 14 showed positive results with traditional microbiological approaches and with PCR-RFLP and FISH analyses. The identified pathogens included the C. neoformans complex (n = 8), Histoplasma capsulatum (n = 2) and Candida albicans (n = 4). Traditional microbiological approaches and PCR-RFLP and FISH analyses showed identical results for the blood culture materials. However, the mean times to diagnosis after the detection of microbial growth in the automated blood culture system were 5 hours, 20 hours and 6 days for detection by FISH, PCR-RFLP and traditional microbiological approaches, respectively. Selected FISH images of the identified microorganisms are shown in Figure 1. The patients presenting invasive mycoses included 9 males and 5 females between 12 and 54 years of age. Nine patients were serologically positive for HIV, with viral loads between 75.098 and 554.987 copies/ml and with CD4^+^ cell counts between 211 and 669 CD4^+^ lymphocytes/ml.

## Discussion

The present work demonstrated the application of FISH for the routine detection of the primary causative agents of invasive fungal infections in patients in a diagnostic laboratory in South America. Molecular methods, including PCR and FISH, have been developed for the rapid identification of fungal agents from primary sample materials. FISH is a comparatively inexpensive and easy-to-perform molecular method that has already been successfully implemented in clinical microbiology for the identification of pathogenic species of Candida and Cryptococcus [13,16,25].

The sensitivity of FISH for fungal infections in CSF proved to be slightly better than conventional microscopy (India ink) under the experimental conditions and allowed the detection of two cryptococcosis patients who were missed by conventional microscopy. Martins et al. [14] also demonstrated that FISH presented better results than India Ink microscopy under their experimental conditions. FISH most likely allowed the detection of fungi cells that had an altered capsular appearance caused by antifungal treatment [26], while India Ink microscopy did not. A multi-centric approach to obtain the numbers of respective samples needed to perform a statistical comparison is now necessary.

Fourteen blood cultures from patients with suspected systemic fungal infections showed positive results. The traditional microbiological approaches and PCR-RFLP and FISH analyses gave the same results in terms of the detection (100% sensitivity) and identification of the fungal agents. However, traditional microbiological assays require the sub-culturing of blood culture samples; microscopic assays, including India ink, lactophenol cotton blue and Gram staining; and biochemical assays, including sugar assimilation, sugar fermentation and enzyme production tests. These assays are slow, requiring 3–10 days and trained microbiologists. PCR-RFLP requires DNA extraction, PCR and electrophoresis to assess the PCR products. In addition, the PCR products must be digested by restriction enzymes, and the RFLP profiles must be evaluated. These steps require at least 20 hours and trained staff with experience in molecular techniques. FISH is robust, easier to perform, and considerably faster, with a time-to-result of up to 5 hours.

In addition to these results, FISH demonstrated the capability of identifying the fungi directly in the biological samples and did not require sub-cultures; these characteristics are essential for conventional diagnoses. This capability is a great advantage for histoplasmosis diagnosis because H. capsulatum cultures should be manipulated under high biosafety containment conditions [27]. Notably, the present study is the first demonstration of the possibility of FISH using combinations of these six adopted probes.

In the present work, 16 blood cultures presented “microbial growth” in the blood culture system but did not present bacterial (Gram stain) or fungal growth (traditional microbiological assays, FISH and PCR). The false detection of microbial growth by blood culture systems has been previously described [28]. The simultaneous lack of detection by FISH and by the other techniques (PCR-RFLP and culture) demonstrated the adequate specificity of the FISH technique.

Regarding the limitations of the present study, we did not use latex agglutination tests to investigate the presence of cryptococcal polysaccharide antigen in the CSF samples. However, Martins et al. [14] developed the Cryptococcus probes that were used in the present work and demonstrated that FISH and the latex agglutination test present similar results when these probes were used. Another limitation is that the combination of probes in this paper did not cover other possible pathogens; however, this limitation was mitigated because the choice of probes used was based on research by Souza et al. [14], who demonstrated the predominant species of fungi that cause invasive mycoses in patients in northern Brazil.

Previous studies have demonstrated that FISH is a useful tool for environmental microbiology studies [23]. The improvement of this technology (the utilisation of enzymes and PNA probes and the association with flow cytometry) motivated the development of several works in clinical microbiology [6,16,17,25,29,30]. Specifically in medical mycology, the detection of Candida spp. motivated most of these studies and resulted in the production of commercial kits [16]. Recently, FISH probes were developed for detecting Cryptococcus spp. [14] and H. capsulatum [21]. Additionally, these studies have clearly demonstrated that the FISH technique is more effective with biological samples that present high fungal cell contents, such as the specimens that were selected for the present work.

This study is the first to assess the detection and identification of important fungal agents, i.e., Candida spp., Cryptococcus spp. and H. capsulatum, using the routine procedures of an infectious disease hospital in South America. These results suggest that FISH is a valuable, robust, fast and easy to perform tool that can be readily implemented in the diagnostic routine of hospital laboratories.
